# Towards dignified healthcare: Patients’ understanding and experiences of respectful and disrespectful care in healthcare facilities in Ghana

**DOI:** 10.1371/journal.pgph.0006135

**Published:** 2026-04-09

**Authors:** Benjamin Abaidoo, Vera Adobea Essuman, Alex Kweku Addo, Monica Getahun, Gordon Amoh, Raymond Akawire Aborigo, Moro Ali, Akye Essuman, Osamuedeme Odiase, Alfred Edwin Yawson, Patience Afulani

**Affiliations:** 1 Ophthalmology Unit, Department of Surgery, University of Ghana Medical School, Accra, Ghana; 2 Eye Department, Korle Bu Teaching Hospital, Accra, Ghana; 3 Departments of Obstetrics, Gynecology, & Reproductive Sciences, University of California, San Francisco, California, United States of America; 4 Family Medicine Department, Korle Bu Teaching Hospital, Accra, Ghana; 5 Research and Development Division, Navrongo Health Research Centre, Ghana Health Service, Navrongo, Ghana; 6 School of Public Health, CK Tedam University of Technology and Applied Sciences, Navrongo, Ghana; 7 Department of Internal Medicine, University of Health and Allied Sciences, PMB 31, Ho, Volta Region, Ghana; 8 Department of Community Health, University of Ghana Medical School, Accra, Ghana; SUPA71 Co., Ltd, THAILAND

## Abstract

Respectful and disrespectful care profoundly influence communication and collaboration between caregivers and patients, shaping outcomes across the continuum of care. Despite patients being the primary users of healthcare services, their experiences of respectful and disrespectful care remain underexplored in Ghanaian healthcare settings. To address these systemic inadequacies, this study explored patients’ understanding and experiences of respectful and disrespectful care. The study employed a qualitative design and was conducted in six healthcare facilities across Ghana. Thirteen focus group discussions were conducted with 90 participants, aged 18–87 years, between October and December 2022. Data were audio-recorded, translated, transcribed, and thematically analyzed. The mean age of participants was 42.7 ± 15.3 years, with a majority (72.2%) being female. Participants associated respectful care with timeliness and quality of patient reception, showing hospitality and friendliness to patients, clarity of communication and patient involvement in decisions, and providing non-discriminatory care. On the other hand, disrespectful care was characterized by long waiting times, poor coordination, poor staff attitudes, privacy violations, neglect of patient priorities, navigational challenges, inadequate infrastructure, and lack of required medicines, equipment, and supplies. These experiences significantly influenced participants’ psychological well-being, satisfaction, and future healthcare-seeking behavior. While respectful care fosters trust and well-being, disrespectful care erodes satisfaction and discourages engagement with healthcare services. Systemic inadequacies must be urgently addressed to create a dignified, inclusive, compassionate, and responsive healthcare system in Ghana.

## Introduction

Respectful and disrespectful care are pivotal in shaping communication and collaboration between caregivers, impacting care delivery across the care continuum and directly influencing health outcomes [1]. These concepts are integral to person-centered care (PCC), an approach that prioritizes patients’ individual needs, preferences, and values [2]. The World Health Organization (WHO) defines respectful care as a fundamental right that preserves patient dignity, privacy, and confidentiality, protects against harm and mistreatment, supports informed decision-making, and ensures continuous care [3]. Respectful and disrespectful care shape communication and collaboration between caregivers and patients, influencing care delivery and health outcomes. PCC is widely promoted by international guidelines, including those from the International Association of Patient Organizations and the Declaration of Patient-Centered Healthcare, which affirm respect for patients’ rights [2–9]. Central to PCC is recognizing patients as partners in treatment decisions, underscoring their right to respectful care [9].

Despite widespread endorsement of PCC and acknowledgment of patients as essential healthcare stakeholders [9], disrespectful and abusive care practices remain pervasive globally [10–13]. Evidence indicates that women, particularly during childbirth, often endure various forms of disrespect and abuse, including neglect, verbal abuse, physical assault, and abandonment [14,15]. Such violations typically stem from systemic deficiencies, including understaffed facilities, inadequate training, insufficient resources, and limited patient education and empowerment [15–17].

The World Health Organization (WHO) defines respectful care as a fundamental right that preserves patient dignity, privacy, and confidentiality, protects against harm and mistreatment, supports informed decision-making, and ensures continuous care [18]. However, the demanding and stressful nature of healthcare environments can often precipitate disrespectful behaviors. Burnout, personal frustrations, and systemic failures among healthcare personnel can manifest in abusive language, disrespectful gestures, or other forms of mistreatment [19–22].

Beyond individual stressors, interpersonal and systemic factors such as communication challenges and entrenched power dynamics significantly contribute to disrespectful care [22–24]. Hierarchies within healthcare settings can undermine team effectiveness, patient safety, and care quality [24]. In such environments, frustrations may lead to disrespectful interactions, which are often redirected toward patients.

Despite its widespread impact, disrespectful care remains largely under addressed in healthcare systems. Historically, many organizations have overlooked this issue, with some tacitly accepting it as part of the norm [19,22]. This inertia is compounded by the difficulty of addressing misconduct from individuals in positions of power, which can deter patients and caregivers from reporting incidents, especially when institutional leadership fails to intervene effectively [1].

Recognizing the gravity of these issues, the WHO has called for further research into the definition and measurement of disrespectful care within healthcare settings [25]. However, consensus on actionable strategies to improve respectful care remains lacking. Addressing this gap will require deeper exploration into respectful/disrespectful care and its impact on patient well-being.

In spite of the fact that several studies have reported the significance of dignity, empathy, and communication in clinical encounters [26–28], up to now, there is no documented systematic investigation on the understanding and experiences of Ghanaian patients about respectful and disrespectful care in healthcare facilities which is an important knowledge gap, as the health system in Ghana continues to expand access to services. Conducting such studies provides necessary evidence to contextualize patient experiences, identify gaps in service delivery, and inform policies aimed at strengthening PCC in Ghana. Also, in Ghana, efforts to improve patient care led to the introduction of the 2002 Patient Charter, which outlines patients’ rights and responsibilities [29]. While this charter was a significant step forward, its operationalization has been inconsistent, and reports of disrespect and abuse persist [30]. The charter highlights the importance of patient needs and preferences, yet addressing disrespectful care comprehensively remains a critical challenge. Understanding patients’ perceptions and experiences of respectful and disrespectful care is essential to developing context-specific interventions. Such insights can uncover nuanced aspects of respect and disrespect that might not be apparent to care providers. This study explored patients’ understanding and experiences of respectful and disrespectful care in selected healthcare facilities across Ghana.

## Materials and methods

### Study design

We employed a descriptive qualitative design [31,32], to explore patients’ understanding and experiences of respectful and disrespectful care in selected healthcare facilities in Ghana. Using a constructivist epistemological approach [33,34], we recognized that patients’ lived experiences and social interactions within healthcare settings co-create knowledge about these concepts. This perspective acknowledges the diverse realities shaped by institutional, interpersonal, and cultural factors in healthcare practice. The constructivist framework guided data collection and interpretation, enabling participants to share nuanced perspectives on care practices and providing contextually grounded insights to inform strategies for improving patient-provider relationships in Ghanaian hospitals.

### Ethics statement

Ethical clearance was obtained from the Institutional Review Boards (IRB) of the University of California, San Francisco (UCSF) (IRB number: 22–36140), and the Korle Bu Teaching Hospital (KBTH) (KBTH-IRB00060/2022) to cover research activities conducted at the Korle Bu Teaching Hospital in the Greater Accra Region. Additionally, ethical approval was secured from the Navrongo Health Research Centre (NHRC) (NHRCIRB 460) to ensure the protection and care of participants recruited from study sites located in the Upper East Region of Ghana. The gap in the renewal process for the ethical clearances was administrative (related to processing timelines at the ethics committee, rather than issues with the study itself). All participants provided verbal informed consent and received an incentive of a bar of soap for their participation. This was done in the presence of an experienced Research Assistant who served as an independent witness. In their preferred language, participants received a thorough explanation of the objective of the study, procedures, possible risks and benefits. The date, time, and witness name were recorded in a secure permission log along with the participant’s vocal consent confirmation. All the Institutional Review Boards (IRBs) examined and accepted the use of verbal consent. Given the study’s setting and participant base, this strategy was judged suitable. Furthermore, the study adhered to the Declaration of Helsinki’s ethical principles for medical research involving human subjects and followed the observation of anti-COVID-19 protocols, as the study was conducted during the COVID-19 era.

### Study sites and participants

The study was conducted at the Korle Bu Teaching Hospital (KBTH) in Accra, the Upper East Regional Hospital, and four district hospitals (Bongo, Paga, Builsa North, and War Memorial Hospitals) in the Upper East region (UER) of Ghana. The KBTH is Ghana’s largest tertiary health facility, serving the health needs of Ghanaians and other nationals from neighboring countries. Eight departments were purposively selected from this facility based on high patient volumes and the presence of functional quality improvement personnel. The Upper East Regional Hospital serves as the region’s main referral facility. All the district hospitals serve the health needs of the people in the districts. Selected facilities were relatively accessible to the study team and represented different levels of health facilities in Ghana.

Eligible participants were adults aged 18 years and above who had used services at the participating health facilities within the past year. We included individual patients and their accompanying caregivers/family members who were willing to provide verbal informed consent for participation. Those who could not communicate in English, Ga, Ewe, Twi, Kasem, Buli or Nankani (main languages spoken in the study settings), including those who could not consent or did not feel well enough to participate were excluded.

### Data collection

Data collection for the study commenced on 15^th^ May 2023 and concluded on 10^th^ January 2024 at the study sites located within the Korle Bu Teaching Hospital (KBTH). For the study sites situated in the Upper East Region of Ghana, data collection began on 7^th^ June 2023 and ended on 8^th^ January 2024. We conducted 13 focus group discussions (FGDs) (eight at KBTH and five in the UER) with 90 participants across the six study facilities. The number of FGDs was guided by the principle of data saturation [35]. In this study data saturation occurred when successive interviews yielded no new themes, categories, or insights relevant to the objectives of the study. We conducted ongoing, iterative analysis alongside data collection through the review of transcripts after each interview to identify emerging codes and patterns. Once we noted redundancy with additional interviews confirming that no novel information was being obtained, data collection was ended. This process guaranteed the documentation of a detailed and adequate presentation of experiences of participants consistent with accepted standards for data saturation in qualitative studies [36]. The FGD guide was developed to explore patients’ understanding and experiences of respectful and disrespectful care and organized around key domains, with open‑ended questions aligned to the study themes to elicit detailed narratives. It was piloted at the KBTH before application in subsequent FGDs. Experienced graduate-level researchers (BA, AKA, GA, RAA, EK and MA) facilitated the discussions. The study participants were recruited using a purposive sampling approach. This sampling strategy was chosen to ensure that individuals meeting the inclusion criteria described above were deliberately included to enable us capture diverse perspectives relevant to the objective of the study while keeping the methodological rigor. This approach is consistent with qualitative descriptive designs, which prioritize the inclusion of participants who can provide rich, contextually grounded accounts of the phenomenon under study [31–34]. Potential participants were first screened for eligibility, and those who met the inclusion criteria were informed about the study objectives and procedures. Those who gave verbal informed consent were invited to participate in the FGDs at a location within the hospital that ensured privacy. Thus, discussions were held in private settings, with only participants and facilitators present, ensuring confidentiality. Facilitators emphasized that there were no right or wrong answers, encouraged honest sharing, and assured participants that responses would remain anonymous.

To ensure social distancing, each FGD was limited to six to 10 people. Before the start of each discussion, we sought permission to audio record the discussion. Members of the research team also introduced themselves to participants, explained the purpose of the study, and stated the ground rules before each discussion. We obtained respondents’ socio-demographic information, including gender, age, marital status, educational background, parity, and occupation. Discussions were conducted in English, Ga, Ewe, Twi, Kasem, Buli or Nankani, as our facilitators and respondents were familiar with these languages. The research team took field notes during each discussion. Each discussions lasted about 60 minutes.

### Data analysis

The audio-recorded discussions were transcribed directly into English, and transcripts were reviewed for accuracy before initiating analysis. We used a thematic analysis approach following steps adapted from the approach proposed by Braun and Clarke [37,38]. Our analysis was iterative using both deductive and inductive approaches. Initially, we read the transcripts to familiarize ourselves with the data collected. Using the interview guides, we deductively developed the initial codebook which was further refined after additional reading of the transcripts. During coding of the transcripts, a team of five researchers inductively updated the codebook with new codes that emerged from further reading of the transcripts, guaranteeing methodological rigor and contextual relevance. Coding was done in Dedoose software version 9.0.17 [39]. We reviewed excerpts for each code to identify themes, which were examined in relation to the entire data set, labelled, and the data under each theme were summarized. We selected representative quotes for each theme to illustrate the findings. We discussed differences in coding or interpretation among the research team until consensus was reached. Where needed, a third reviewer provided arbitration. Reliability was strengthened through double‑coding, regular team debriefs, and adherence to a structured coding framework aligned with the study’s domains and themes. The demographic characteristics of participants are summarized using means and standard deviations for continuous variables and percentages for categorical variables.

### Reflexivity and rigor

All the authors (eight male and four female) are trained in qualitative interviewing, thematic analysis, and writing. The authors hold advanced academic and professional qualifications, including MSc, MD, and PhD degrees. Each member had prior experience in public health, social, and clinical research. As a group of researchers from Africa based in Ghana and the US, including clinicians and non-clinician researchers, we recognize that our backgrounds influence how we interpret data. We therefore maintained reflexivity and rigor in this study by clearly explaining our methodological processes, findings, and conclusions to ensure the reliability of our findings [40–42]. The research team had no prior relationship with any study participant. We explained the processes involved in data collection and the analysis plan to ensure the dependability of our study. An independent researcher reviewed the transcribed data and the field notes to ensure the transcriptions were done well and to ensure the study’s confirmability. To ensure the transferability of our findings, we provided a sufficient description of the data collected and our study participants’ demographic background to enable readers to assess the transferability of our findings. Credibility was ensured by engaging all the authors to review the transcripts and provide feedback on the manuscript drafts. To improve the transparency of our results, we used the Consolidated criteria for reporting qualitative studies (COREQ) standard of reporting qualitative studies as a guide in reporting our findings.

## Results

### Socio-demographic characteristics of participants

The mean age was 42.7 ± 15.3 years, with minimum and maximum ages, 18 and 87 years. Most participants were females (65, 72.2%), married (61, 67.8%), and had attained secondary/technical education (42, 46.7%). Most (52, 68.5%) respondents had 0–3 children with an average parity of 2.6 ± 1.9. Most participants (54, 60.2%) were from the Korle Bu Teaching Hospital (Table 1).

**Table 1:** Socio-demographic characteristics of the participanTS IN THE FOCUS GROUP DISCUSSIONS.

Socio-Demographic characteristics	Number (n = 90)	Percentage (%)
Sex:		
Male	25	27.8
Female	65	72.2
Marital status:		
Married	61	67.8
Single	24	26.7
Divorced	3	3.3
Never married	2	2.2
Education:		
Never been to school	14	15.6
Primary	20	22.2
Secondary/technical	42	46.7
Tertiary	14	15.6
Parity:		
0-3	52	68.5
4-6	21	27.6
> 6	3	3.9
Occupation:		
Unemployed	11	12.2
Civil/public servant	11	12.2
Farmer/artisan	16	17.8
Trader	22	24.4
Others	30	33.3
Facility:		
Korle Bu Teaching Hospital	54	60.2
Bolgatanga Regional Hospital	7	7.8
Bongo District Hospital	7	7.8
Builsa North Hospital	7	7.8
Paga District Hospital	10	11.1
War Memorial Hospital	5	5.6

The mean age of participants = 42.7±15.3 years. Mean parity = 2.6±1.9.

### Emerging themes and sub-themes from the interviews

We organized our findings under four themes: patients’ understanding of respectful/disrespectful care, patients’ experiences with respectful care, patients’ experiences with disrespectful care, and the impact of disrespectful care, each with several deductive sub-themes (Table 2).

**Table 2:** Themes and sub-themes generated from the focus group discussions.

Themes	Sub-themes
Patients’ understanding of respectful/disrespectful care	1. Timeliness and quality of patient reception. 2. Showing hospitality and friendliness to patients. 3. Clarity of communication and patient involvement in decisions. 4. Providing non-discriminatory care.
Patient’s experiences with respectful care	1. Being welcomed and spoken to politely. 2. Empathy and responsiveness to individual concerns. 3. Good communication and Patients’ engagement in care. 4. Friendly interactions with providers.
Patient’s experiences with disrespectful care	1. Long waiting times. 2. Poor coordination with scheduling. 3. Lack of privacy and confidentiality. 4. Poor attitude of support staff. 5. Poor health facility infrastructure. 6. Challenges navigating location of different services. 7. Lack of required medicines, equipment, and supplies.
Impact of respectful/disrespectful care	1. Psychological effect on patients. 2. Satisfaction with service delivery. 3. Impact of future health seeking behaviour.

### Patients’ understanding of respectful/disrespectful care

Patients described respectful care as timely and respectful reception on arrival, showing hospitality and friendliness to patients, communicating effectively and involving patients in care decisions, and providing non-discriminatory care. Conversely, they described disrespectful care as one that is characterized by a lack of clear communication, an unwelcoming demeanor, and perceived indifference, leaving patients confused and undervalued.

#### Timeliness and quality of patient reception.

Patients often associate respectful care with healthcare workers who are prompt and attentive upon their arrival at the Outpatient Department (OPD), indicating a strong preference for such proactive engagement. This explains the significance of a warm welcome upon the arrival of patients. It involves prompt acknowledgment, polite communication, and clear directions, and empathy amidst delays in service provision. Timely and respectful reception modifies the first impression of patients, trust, and confidence in the provision of healthcare services.

*“…..Those who normally run to patients to treat them on arrival at the OPD are those who are showing respectful care. Most patients are looking for such health workers to treat them when they visit the hospital.” SDH- FG7*

*“In my view, respectful care is to give a patient the utmost attention and ensuring that you go all round to make sure the patient is better. In respectful care, you don’t feel like this patient is inconveniencing you or you are the boss and without your help the person will die, or the person has no option.” KB-EY-FG5*

*“When you are sick and you go for treatment in a health facility, it is just like carrying a heavy load to be off-loaded. ……. If they are able to welcome you nicely by listening to you and accepting you and treating you with respect the pains always reduce, and you will know that you have come to a facility that provides respectful healthcare.” SDH-FG6*

In contrast, participants noted poor reception on arrival and delays in receiving care as disrespectful care. Some reported a lack of respect for their time when they had to wait too long to be seen or when their appointments were cancelled after they had made plans to be at the facility.

*“Few health workers demonstrate respect to patients. Patients sometimes are afraid to ask for help because some of the caregivers do not talk politely to them. Some delay in attending to the needs of patients. There was a time a patient’s water got finished and a nurse was called to come and attend to her needs. The nurse delayed before attending to the patient. So, where is the respect? If they call you to come and attend to a patient, you have to run and come. That is why I said they don’t show respect.” BDH-FG6*

*“Nurses in this hospital don’t show respect because my brother died here and was left there for some time. They were supposed to take him out of that room and report on his death. Instead, they just left him there till evening before the mortuary men came and pick him up. So, I see that nurses in this hospital don’t give respect.” BDH-FG1*

In addition, they noted it was disrespectful when they arrived and were not received well and promptly, their needs were not prioritized, and providers were inattentive to their preferences. They noted some providers were usually busy on their phones or with other things when they should be paying attention to them. This was especially frustrating when they brought patients who needed assistance, and providers did not immediately come to help them.

*“If a patient is here and no one has time for the person, it is disrespectful care. That is not giving the patient the needed attention.” KB-CAD-FG1.*

*“Disrespectful care can also be a situation whereby a staff taking care of you ends up being busy with the phone, taking a ‘selfie’, or dressing their hair. Meanwhile, he/she [health worker] is expected to concentrate on the work.” BDH-FG6*

#### Showing hospitality and friendliness to patients.

Hospitality and friendliness were noted as core to respectful care. It captures the perception of patients about interpersonal warmth and attentiveness as a significant aspect of respectful care. Hospitality and friendliness are not superficial gestures but represent the broader philosophy of PCC where patients feel welcomed, valued, and treated with dignity. In the Ghanaian healthcare context, such behaviors are important as they build patients’ trust in providers and influence willingness to seek care and adhere to prescribed treatment regimen. In this study, showing hospitality and friendliness to patients was done through actions such as greeting patients warmly, using polite language, offering assistance with navigation through the facility, and showing patience when explaining procedures. Patients consistently highlighted how such care makes them feel valued and happy to return for treatment.

As one patient shared, *“I have a hospital nearby my community, but because of the care I am receiving here, I always want to come here for my care. In respectful care, caregivers show hospitality to patients, and patients are always happy to come.” KB-PC-FG2.*

Another respondent underscored the importance of friendliness, being hospitable, and being attentive to patient needs in nurturing positive interactions, patient satisfaction, and fostering a respectful care environment.

*“It is a kind of care that brings about satisfaction as a result of the friendly and hospitable behavior of caregivers. Whenever I come here, I am treated well. Respectful care is where the caregiver will have patience and spend time on things that matter to the patient.” KB-PC-FG7*

On the other hand, not being shown hospitality and being unfriendly indicated disrespectful care. This included not being offered a place to sit, not being given clear directions, and providers indifference about their situation.

*“Please I have realized that when they finish checking your vitals and blood pressure, they don’t tell you where to sit so sometimes we get confused as where to sit or go next. So for me, I expect them to give further directions after checking our vitals and blood pressure. The processes should be well explained. If not explained well, then I see it as disrespect.” KB-MD-FG7*

*“There are situations where patients are weak and unable to walk. Yet, you may see health workers sitting down and looking at the patient. When you struggle before sitting down, they [health workers] will not ask you about your condition but will rather sit down making calls until they are done with whatever they were doing before attending to you. After asking you, they will even frown and sit there.” SDH-FG4*

#### Clarity of communication and patient involvement in decisions.

This includes listening to patients attentively and explaining procedures in a manner that the patient understands, and addressing them courteously. It also includes their involvement in care decisions which implies respect for their autonomy through informed consent, discussion of treatment options, and valuing of their preferences. Participants noted the importance of providing sufficient information in a respectful manner and involving patients in care decisions. They noted this creates an opportunity for a partnership between the provider and the patient in the planning of care and demonstrates respect to them.

*“Respectful care is having interaction with the patient concerning the sickness and giving the patient the needed information and allowing the patient to contribute. It is involving the patient in the care and not ignoring the interest of the patient.” -KB-CH-FG1.*

*“What they did for me is that they explained to me things I do not know and asked me if I have something to say. This shows that they treated me with respect and involved me in their care plan.” -BDH-FG10.*

Disrespectful care, on the other hand, was also viewed in its broader sense as poor conduct of the provider during communication with patients. They explained that communicating with patients in an impolite manner, being verbally abusive, or in a manner that is not culturally and linguistically appropriate, is an act of disrespectful care.

*“Frankly speaking, most health workers treat patients with disrespect. They don’t know how to talk to patients, be it an elderly or younger patient. So, when health workers are not polite to patients, they are not treating patients with respectful care.” BRH-FG6*

#### Providing non-discriminatory care.

This theme underscores the importance of treating all patients equally, regardless of age, gender, socioeconomic status, ethnicity, or health condition. This was seen in fair treatment, equal attention, and unbiased communication. Participants also noted that respectful care should not be discriminatory, and that everybody should be treated the same. This was particularly related to how they were received on arrival at the facility and the order in which they were seen. The role of the front desk in cultivating respectful care was considered very important and therefore discriminatory care in that department was thought to be disrespectful.

*“As my sister said, there shouldn’t be any discrimination. If you go to a church, the first person you meet is the usher. The reception you will receive from the usher will tell you how the church looks like and whether you will stay there or not. The front desk at the clinic serves as the ushering unit of the clinic and their reception matters a lot to us.” KB-EY-FG2*

### Patient’s experiences with respectful care

Respondents reported experiencing both respectful and disrespectful care in the facilities. Descriptions of respectful care were related to: good reception, respect for values, choices, and needs, patient’s engagement in care, and friendly communication from clinical staff.

### Being welcomed and spoken to politely

Being welcomed and spoken to politely shows the importance of welcoming patients warmly and respectfully upon arrival. Such practices were perceived as fostering dignity and trust in care providers. Respondents described various experiences representing a good reception at the facility. Such good reception included being welcomed nicely, being directed to the right place, and being spoken to politely, which improved the interaction between the patient and the provider. It also played a significant role in leaving a good impression of patients about the healthcare facility.

*“In this facility, the first time I came here, I was given a good reception, it was very nice. The nurse directed me nicely and explained to me that they are here because of me, because we are their customers which I think is part of the healing process.” KB-CAD-FG2*

*“Because of the respect I am given at the OPD, it makes me feel that I will get my healing here. The way you talk to us also makes us feel we will be well.” KB-CAD-FG5*

*“They take care of us well, right from the OPD to the doctors and nurses, they make us feel fine because of the manner they speak with us. It makes us feel satisfied. In fact, the way the care giver talks and the advice they give to you makes you feel that the person is interested in your recovery.” KB-CAD-FG1*

### Empathy and responsiveness to individual concerns

This explains the need to listen attentively to patients, acknowledge their concerns, and adapt care to their personal needs. Patients noted they had experienced respectful care in the form of providers empathizing with them and ensuring their needs, values, and choices were respected. This took different forms, including providers discussing the cost of medications before prescribing to ensure they could afford them.

“*…….. Concerning the drug aspect, to me the doctor I saw explained everything to me. He said the medicine is expensive (about GHC1000 and above) and asked if I can afford it. To me I realized he was much concerned about me. He told me what the medicine can do as well.” KB-CAD-FG6*

*“I think our values are respected anytime we visit this facility but we will not get it done a hundred percent due to some patient’s and health worker’s characters.” BDH-FG2*

Others, however, noted a lack of respect for their needs and values, especially when providers did not entertain their preferences and choices.

*“It is not all the time that our choices are respected because sometimes you can tell the providers your choice but they will say that your choice will not work. Sometimes it depends on the kinds of sickness that we are suffering from.” SDH-FG7*

#### Good communication and patients’ engagement in care.

This includes clear and respectful communication and active involvement of patients in decision making. Thus, patients valued attentive listening, understandable explanations, and shared decision‑making. Several patients noted that providers explained things clearly to them to understand their diagnoses and treatment, and engaged them in decisions regarding their care. Patients appreciated such engagement and noted it indicated respectful care.

*“What they usually do is that anytime I do not understand something pertaining to my treatment they explain things to me and show me what to do for my child. This shows that they treated me with respect and involved me in the care.” BDH-FG10*

*“For today’s treatment the doctor who took care of me, was very good and respectful because he engaged me in communication concerning my health, and life and even extended it to my family members. So, what I will say is that God should richly bless him and his work will improve so that he will continue to treat patients with respectful care and involve them in the treatment plan.” SDH-FG5*

Some patients, however, noted that providers did not always explain the purpose of medications to them.

“*I think it is friendly and respectful. My only problem is when it comes to medication, some of the doctors only write it for you without explaining it.” KB-CAD-FG1*

#### Friendly interactions with providers.

Many participants also indicated that providers interacted with them in a friendly and respectful manner. This interaction extended beyond clinical providers to non-clinical staff such as the security men.

*“In this facility, there is respectful care. I remember when I was asked to go for ECG scan, I didn’t know there even though I come here often but because the facility is big, I didn’t know where to find it. I went to a security man to ask him and he left his post to accompany me to the place, so I see that he has given me respect. The doctor I met was good because of the way she spoke to me and received me. This shows respect is practiced here.” KB-AN-FG6*

*“When I came here, I realized that there was respectful care. Doctors patiently explain things to you and give you the help you require. The nurses support a lot in the care of your child. They inform you about what will help your child to receive quick recovery and things that will not help the child. I am happy here.” KB-CH-FG1*

### Patient’s experiences with disrespectful care

Patient’s experiences of disrespectful care included the following: long waiting times, lack of privacy, poor attitude of support staff (communication issues and discrimination), poor environmental conditions, and poor access to required health services/pathway (lack of resources such as photocopy machine and medicine supply).

#### Long wait times.

This demonstrates the frustration of patients with extended delays before receiving care. Long waits, often without explanation, were perceived as disrespectful and discouraging. Long waiting time was the most common complaint among respondents, which they perceived as important in determining service quality. Long waiting times occurred during pick up of patient folders, measuring of vital signs, consulting the doctor, receiving medications, having and receiving laboratory tests, and paying for services. Some patients perceived this as disrespect for their time, given that they often left home very early to come to the facility, yet end up spending the whole day at the clinic. Sometimes they are even unable to see the doctor.

*“Long waiting hours. Sometimes you will come early and the doctors will not come on time so you have to wait for long. I am from the Volta region and I have to set off around 1:00 am and arrive early just to be waiting for the doctors. So sometimes it is frustrating.” KB-AN-FG7*

Participants noted discriminatory practices that further worsened the waiting times such as people who came later being seen earlier for several reasons including paying money to the records staff.

*“At the OPD (the records and front desk), they give us numbers, but they hide some of the numbers to be given to some special people of their choice. Three days ago, I saw that those who came late were told that the afternoon shift doctors will take care of them. But all of a sudden, the nurse took a number from the folder and gave it to a certain woman who came in late. So, the patient close to me started complaining then I told the person to exercise patience because it could be that the person’s illness is a very serious one. I went to inform the nurse that next time she should explain certain actions like this to patients so that they don’t get offended. The nurse thanked me for my advice.” KB-PC-FG5*

*“When I am coming here, I come very early. There are some people who come to meet you but before you realize they have been seen. There was a man beside me and I asked him why are those people who came to meet us seen earlier? The man replied that here if you want to be seen on a particular day, you need to come a day before to tip those who prepare the cards (records). That was what he told me because he confirmed that he did that. I told him that I can’t pay someone who is being paid.” KB-CAD-FG7*

#### Poor coordination with scheduling.

This shows the dissatisfaction of patients with disorganized appointment systems and unclear scheduling processes. This is usually observed in missed or overlapping appointments, lack of communication, and inefficient coordination which leads to frustration, wasted time, and reduced confidence in the healthcare system. Patients also complained about appointments being rescheduled without respect for their time and where they are coming from. For example, they said someone might already have taken their leave because of a scheduled surgery, only for it to be cancelled on the day of the surgery. Sometimes, the appointments were cancelled and not communicated to them, and they still come to wait for several hours before being told to go home.

*“I think in my experience, our time too is not respected. There was a day I was scheduled to have a surgery so I took my annual leave. I live in the Bono East region of Ghana. I was later called that the surgery has been cancelled and the new date will be communicated but I needed to be reviewed before the surgery will be done. I came for the review and the doctor said, you are going to have the surgery next two days. Meanwhile, I had already cancelled my annual leave and I wasn’t prepared for the surgery at that time. So, I think our time too is not respected.” KB-EY-FG5*

*“They know that there are times that doctors will not be available to attend to you yet they will tell you to come early in the morning and wait for long hours before you are later told to go home because there is no doctor. It hurts so much.” KB-MD-FG2*

#### Lack of privacy and confidentiality.

This sub-theme accentuates the concerns of patients about inadequate protection of personal information and private interactions. Breaches to this included discussing sensitive issues openly or exposing patients during care. Several respondents reported a lack of privacy during consultations. This included other people walking in during consultations as well as providers discussing their problems openly in a way that other patients could overhear them.

*“…….. Again, our privacy is not considered because sometimes you can be with a doctor, and while the doctor is attending to you, someone just walks in, which is not the best. They normally do that here, so you need to let them know so that they can put a stop to it.” KB-PC-FG4*

*“Unfortunately, most of the consulting rooms in this facility are open with no consideration to patients’ privacy. All the things and secrets you discuss with the provider may sometimes be heard by other patients. There is no privacy in this facility. I think this is not the best of practice.” KB-EY-FG3*

#### Poor attitude of support staff.

Poor attitude of support staff reflects the concerns of patients about negative interactions with some of the non‑clinical staff. Rudeness, indifference, or lack of assistance created feelings of disrespect and frustration. Participants reported poor attitudes of both clinical and support staff in various forms, including rude communication and discrimination during care provision. In KBTH, there were especially many complaints about support staff at the records section of the OPD, who were noted to be rude, discriminatory, and talked to patients in an annoyed tone. Similarly, some noted painful experiences with nurses who were so rude that the patients wanted to leave the facility without receiving care.

*“I came here one day and my temperature was measured and it was high. The nurse then spoke to me rudely by yelling at me that my temperature is high so I should go and sit outside. She added that you can’t see the doctor if your temperature is high. I waited outside and even at a point I thought of going home. In fact, I almost wept. At a point, I had the feeling that I should go to surgical to check my temperature. I didn’t know anyone there. I went to meet a nurse there and I asked her to check my temperature and she did and it was down so I returned to the first person. Then she also checked my temperature and saw that it was down so she asked me to go inside. This was during the COVID 19 time.” KB-CAD-FG5*

*“The way they work or how they welcome patients is not usually done right, because of this, there is no respect.” PDH-FG5*

#### Poor health facility infrastructure.

This describes the concerns of patients about inadequate physical environments, including overcrowded wards, broken equipment, poor sanitation, and limited resources which undermines the dignity of patients and confidence in care. Poor environmental conditions and deficiencies in the healthcare system which does not conform to appropriate standards of quality care were identified as experiences of disrespectful care. These included a lack of and dirty toilet and bathroom facilities, having to pay to use toilet facilities, and inadequate or uncomfortable seats in the waiting areas. Respondents in one facility in the UER described a disturbing situation where patients had to defecate in the open, including on people’s farmlands, and being forced to collect it when apprehended by the owners.

*“Our problem is toilet facilities; we don’t have toilets. We don’t also have bathrooms so we have been doing open defecation. Sometimes you would go and defecate in someone’s farmland and the person would say hey, hey, hey……clear your toilet. At times, you wouldn’t want to clear the toilet with your hands because you have come to the hospital. As for the bathing when you come you have to go to your nearby friend’s house and bath and if your patient’s case is so serious, then she will not bath for several weeks.” BDH-FG5*

*“Please I would like to know if it is compulsory to pay for visiting the urinal outside. Because some patients may not have much money on them. At the hospital where I had my referral, patients are not allowed to pay for using the urinal.” KB-OG-FG2*

*“My problem has to do with where we sit. Sometimes, when you come you see pregnant women standing whilst some husbands and children are rather sitting. When you tell them to excuse you to sit then they will give you cheeky responses. I would be pleased if they are informed about it to allow the patients to sit.” KB-OG-FG4*

#### Challenges navigating the location of different services.

Challenges navigating the location of different services is about the experiences of difficulties in finding various departments or services within hospital. Lack of clear signage, poor directions, and confusing layouts caused frustration and delays. Several participants reported finding it hard to navigate the health facility, especially in KBTH, which is quite big. This was compounded by the staff being rude when they asked for directions.

*“There are times you may forget the clinical processes because you might have visited the hospital for long. So, while finding out about the processes, some of the nurses can be so rude to you.” KB-MD-FG3*

***“*** *There was a time I was asked to do some labs so I asked for direction to the place because I didn’t know the hospital very well. The person who directed me couldn’t direct me very well so I got lost and had to come back to the clinic and when I took a look at the time it was 12:00 pm and was directed to a different place to have the labs. When I returned from the lab to the clinic it was 3:00 pm. In fact, that day I became very tired because of the walking. At the clinic, the person who attended to me also left me and did not inform me that he was done with me. Because what usually happens after you have been attended to is that, you are asked to pay a fee so since that fee was not collected, I assumed that the person has not finished with me. Later, the person came back to apologies for not informing me he was done. Because of the incidence I left here almost at 5:00 pm.” KB-OG-FG2*

#### Lack of required medicines, equipment, and supplies.

This sub-theme explains the frustrations by patients in situations of shortages of essential medicines, equipment, and supplies. Inadequate resources disrupted care, delayed treatment, and undermined trust. A key challenge mentioned by several respondents, which influenced their experience, was the lack of medications prescribed, such that almost every medication had to be bought from outside the facility. In addition, some patients perceived that providers were hoarding the medications, such that it was not available from the pharmacy but could be bought from them. Others also reported on a lack of equipment, such as ultrasound machines.

*“They should make sure there is medicine in the hospital so that when you bring your patient, they will get medication for the patient. Sometimes, there are some drugs that should not get finished in the hospital but those medicines sometimes get finished in the hospital unless you go and buy them outside the hospital, this is what I also have to say.” PDH-R6*

*“The challenge is that; the hospital lacks medication supply. …… Some of the nurses are selling these medicines so when your patient needs a particular medicine, they will tell you that they have the medicine so you can buy from them.” BDH-R5*

Further, respondents reported frustrations of not finding photocopiers within the facility when they were asked to make copies of their documents.

*“Three days ago, after I had been attended to, I wanted to do photocopy and I searched everywhere in Korle Bu but I didn’t get one so I had to go to the road side to do it. This is not fair. It is quite frustrating to me. Korle Bu is a big place so I suggest that at every point we should have a photocopy machine to enable us leave early when we come.” KB-OG-FG6*

### Impact of respectful/disrespectful care

Respondents identified three interrelated impacts of respectful/disrespectful care: psychological impacts, satisfaction with care, and impact in future care seeking.

#### Psychological effect on patients.

This explains how disrespectful care practices negatively affects the mental well‑being of patients. This included feelings of anxiety, frustration, and loss of dignity. Respondents reported several positive psychological effects of respectful care on patients, including building their own confidence and confidence in the provider, calming them, reducing their pain, making them feel valued as a person, and giving them hope that they will be healed.

*“Respectful care has a positive psychological effect on you the patient. Because it has formed something in your mind in such a way that you feel like this doctor when I see him, the way he will speak with me and comfort me, that alone will heal me. ….., whenever I come and see the doctor the sickness vanishes because of the presence of the doctor.” KB-PC-FG4*

*“Respectful care will let you calm down and the pain will reduce. It makes you feel that you are really being treated well and comforted. When you are respected, it means you have been counted as part of human beings.” SDH-FG4*

On the other hand, disrespectful care had negative psychological impacts such as increased anxiety, worry, and discouragement, which they perceived could aggravate the patient’s condition.

*“With disrespectful care, when you come to the hospital and you are not given the attention you need, you become worried and that alone can make you feel the sickness the more. There was an instance, when I left the OPD to eat because I was hungry. I came back to inquire if my name was mentioned in my absence, and the response I received from the nurse was ‘where were you when your name was mentioned.’ This was said in a rude manner.” KB-CAD-FG5*

*“What the health workers are doing definitely creates problems and worries because, it has become the perception of patients that in this hospital whether you come early or not, you will not see your doctor or provider early.” BDH-FG1*

#### Satisfaction with service delivery.

Satisfaction with service delivery explains the overall impression of the patient about the healthcare experiences. In this study, it was connected to respectful communication, timely reception, empathy, and adequate resources. Respectful care was also noted to increase patient satisfaction with care, especially when their expectations were met or exceeded. This further increased the psychological impacts noted above.

*“When a doctor is treating you with care and respect, it gives you good health and you become satisfied with the service delivery. ……..And if the doctor talks to you and shows you all that you need to do in order to help you, it always makes you feel very happy because the doctor will tell you that if you take the medicine your sickness will go.” BDH-FG7*

In contrast, disrespectful care led to dissatisfaction with the services, which reduced trust and confidence in the healthcare provider.

*“A disrespectful care brings about dissatisfaction so much that at a point I decided not to visit the hospital again and that whatever will happen should happen.” KB-PC-FG7*

#### Impact on future health-seeking behaviour.

This explains how the experiences of patients influence future care decisions. Respectful treatment, empathy, and effective communication encouraged continued use of services. Respondents noted that when they received respectful care, they would come back to the facility again when they were not well in the future, while disrespectful care would not make them return.

*“With respectful care, you always feel like going to the hospital even when you are not well. Whereas when there is no respect or it is a disrespectful care, you feel like not attending the hospital. Because when you feel like when you go, you will be shouted at so I shouldn’t attend.” KB-PC-FG1*

*“Respectful care brings cure, comfort and satisfaction with service delivery. You won’t go elsewhere apart from that place you received that respectful care. That’s how come I travel all the way from Kasoa to this facility.” KB-PC-FG6“It affects us negatively and creates much worries because since they don’t treat us well, we will not feel like going to the hospital.” BDH-FG5*

## Discussion

This study utilized a qualitative explorative design, incorporating data from 13 focus group discussions to examine patients’ understandings and experiences of respectful and disrespectful care across select healthcare facilities in Ghana. This design was used as it best conveys patients’ nuanced perceptions, lived experiences, and contextual meanings of care practices. From the perspective of the patients, respectful care is associated with timely and courteous reception, hospitality, and friendliness. They further explained that effective communication, patient engagement, empathy, attention to needs and preferences, and nondiscriminatory treatment are integral part of respectful care. Conversely, disrespectful care was linked to system-related challenges (long waits, poor coordination, navigational challenges, inadequate infrastructure, and lack of required medicines, equipment, and supplies) and staff-related (poor staff attitudes, privacy violations, and neglect of patient priorities) challenges. According to the patients, respectful care promotes psychological well-being, satisfaction, and future healthcare-seeking behavior, emphasizing the need for humanized and accountable healthcare service delivery. These findings provide valuable insights to healthcare providers and policymakers regarding what fosters feelings of respect or disrespect among patients accessing health services.

Our findings align with global studies documenting the prevalence of respectful and disrespectful care across healthcare facilities [10–14,43,44]. Variations in patients’ perceptions of respectful and disrespectful care across facilities were evident, likely influenced by cultural backgrounds. Culture shapes individuals’ worldview, self-perception, and interactions [45], making respectful care a concept that can vary significantly across communities and regions. For some patients, respectful care meant a welcoming and timely reception or hospitality, while others emphasized involvement in decision-making during care delivery. Although these definitions reflect general understanding of respectful care, cultural differences remain a critical factor shaping these perceptions [46]. Also, Bridges et al. [47] highlighted the importance of inclusivity in care delivery, with patients feeling respected when properly welcomed and involved in decisions about their care. Similarly, Thompson et al. [48] and Attree [49] emphasized the role of effective communication and timely provision of information in fostering respect. These findings underscore that respect for human dignity is a universal principle across cultural settings.

A respectful reception upon arrival at a health facility was identified as critical to forming lasting positive impressions about the facility. Front desk personnel act as the “face” of the institution, and their hospitality and conduct create a sense of hope and safety for patients. Respect is fundamental to building genuine relationships and effective communication between providers and patients. Kant’s philosophical view of respect as recognition of the inherent dignity of all persons, regardless of background, resonates with this study’s findings [50]. Although patients from diverse ethnic groups participated, this study did not delve into cultural dimensions of respectful care—a topic worthy of further exploration in future research.

Disrespectful care was widely recognized as impolite communication, neglect of patient priorities, and disregard for patient dignity. These findings are consistent with studies conducted in other settings, such as Tanzania and Zimbabwe, where patients reported poor communication, lack of courtesy, unfriendly behavior, and negligence as key elements of disrespectful care [14,51,52]. Health facilities across Asia and the Middle East have also reported physical and verbal abuse, including shouting, scolding, and yelling [53,54]. A particularly disturbing form of disrespect highlighted in this study was health workers’ use of mobile phones while attending to patients—an issue that undermines patient trust and should be strictly addressed through education and accountability measures. When phone usage is justified for work-related purposes, patients should be promptly informed to mitigate misunderstandings.

Patients’ experiences of respectful care in this study reflected timely reception, respect for their values, choices, and needs, engagement in care planning, and friendly communication. Similar findings have been documented by Beach et al. [55] and Dickert and Kass [56], who emphasized that involving patients in decision-making and recognizing their individuality constitute respectful care. Patients in healthcare settings have the right to dignified treatment that respects their safety, privacy, and cultural values, while also allowing them the autonomy to decline suggested actions or seek second opinions. Advocacy groups and public health education campaigns in urban areas may also contribute to differences in awareness of patient rights. Although Ghana’s Patient Charter outlines these rights [29], its operationalization remains poor in many health facilities, as noted by Yarney et al. [30].

Privacy emerged as a critical component of respectful care, with inadequate infrastructure—such as a lack of screening curtains and private rooms—compromising patient confidentiality and negatively affecting patients’ psychological comfort and trust in healthcare providers. Similar findings were reported by Mwasha et al. [14], who identified lack of privacy, poor communication, and disregard for patient needs as key elements of disrespectful care. Respectful care also requires equitable treatment regardless of personal beliefs, ensuring dignity and fairness in service delivery.

Long waiting times were a common source of patient dissatisfaction which is consistent with global evidence linking extended waits to frustration and reduced trust in healthcare systems [57,58]. Negative staff attitudes compounded disrespectful care, with numerous grievances documented against health workers for mistreatment, neglect, and carelessness [49,59–62]. The finding that some staff overlooked critically ill patients is a concern that requires urgent attention to ensure adherence to ethical standards in healthcare delivery.

Environmental conditions, including poor sanitation and infrastructure, also contributed to disrespectful care. According to the World Health Organization (WHO), 10% of healthcare facilities globally lack basic sanitation, with the situation being particularly dire in low- and middle-income countries [63]. Inadequate water, sanitation, and hygiene services impede staff productivity, increase infection rates, and undermine patient dignity [64]. Addressing these gaps is essential for achieving the Sustainable Development Goals (SDGs) by 2030 [65].

Access to essential services, such as laboratory tests and health insurance documentation, was another challenge reported by patients. The Institute of Medicine defines access to healthcare as the timely use of services to achieve optimal health outcomes [66,67]. Barriers to accessing basic services can lead to inequities and unfavorable outcomes, underscoring the need for improved healthcare infrastructure and service delivery.

Respectful care positively impacts patients by fostering psychological well-being, satisfaction, and trust in the healthcare system. Patients who feel respected report better clinical outcomes and greater overall satisfaction [53,66,67]. Respectful care also promotes patient autonomy and active participation in decision-making, enhancing feelings of dignity and empowerment [68–70]. Conversely, disrespectful care erodes trust and often leads to dissatisfaction, anxiety, and reluctance to seek future care [71–73]. Studies from Asia, the Middle East, and Zimbabwe have documented how disrespectful behaviors, such as yelling or abusive conduct, deter patients from seeking care, thus contributing to adverse health outcomes [49–52].

### Strengths and limitations

This study’s strengths include the involvement of the entire research team in data collection, analysis, and interpretation, enhancing rigor and reliability. The inclusion of diverse hospitals and departments from two regions provided rich, contextually grounded insights into patient experiences, as this diversity captured a wide range of patient experiences across different settings and care contexts. Focus group discussions allowed for dynamic exchanges and uncovered nuanced perspectives. However, limitations include potential social desirability bias and groupthink, which may have influenced responses leading to the provision of overly favorable or conforming responses. Institutional and regional variations may affect the transferability of findings, as findings may not fully represent experiences across all healthcare settings in Ghana. The use of and the qualitative approach limits generalizability beyond the study settings but was prioritized for its depth, offering valuable, nuanced insights into PCC by capturing patients’ experiences and perceptions. Despite these limitations, the study offers valuable insights into person-centered care in Ghana’s healthcare system.

## Conclusion

This study highlights critical insights into patient perceptions of respectful and disrespectful care in Ghanaian healthcare facilities. Respectful care, characterized by timely reception, empathy, and effective communication, fosters trust, satisfaction, and psychological well-being. In contrast, disrespectful care—marked by neglect, poor infrastructure, lack of timely care, and negative staff attitudes—undermines trust and discourages future care seeking behavior. These findings underscore the urgent need for humanized, accountable service delivery supported by interventions such as staff training in respectful communication, policies ensuring patient privacy and autonomy, and institutional accountability mechanisms to monitor and address disrespectful practices.

## Supporting information

S1File COREQ checklist. (DOCX)

S2 File Raw dataset of responses from respondents. (DOCX)

S1 Checklist Checklist. (DOCX)
