# Supplementary material for: Towards dignified healthcare: Patients’ understanding and experiences of respectful and disrespectful care in healthcare facilities in Ghana
Source: PLOS Glob Public Health. 2026 Apr 9;6(4):e0006135. doi: 10.1371/journal.pgph.0006135 (PMC13065053; doi:10.1371/journal.pgph.0006135)
Supplement: S2 File — (DOCX) [file pgph.0006135.s002.docx]

**QUOTES FROM RESPONDENTS IN THE FOCUS GROUP DISCUSSION ON RESPECTFUL AND DISRESPECTFUL CARE**

*“…..Those who normally run to patients to treat them on arrival at the OPD are those who are showing respectful care. Most patients are looking for such health workers to treat them when they visit the hospital.” SDH- FG7*

*“In my view, respectful care* *is to give a patient the utmost attention and ensuring that you go all round to make sure the patient is better. In respectful care, you don’t feel like this patient is inconveniencing you or you are the boss and without your help the person will die, or the person has no option.”* *KB-EY-FG5*

*“When you are sick and you go for treatment in a health facility, it is just like carrying a heavy load to be off-loaded. ……. If they are able to welcome you nicely by listening to you and accepting you and treating you with respect the pains always reduce, and you will know that you have come to a facility that provides respectful healthcare.”* *SDH-FG6*

As one patient shared*, “I have a hospital nearby my community, but because of the care I am receiving here, I always want to come here for my care. In respectful care, caregivers show hospitality to patients, and patients are always happy to come.” KB-PC-FG2.*

*“It is a kind of care that brings about satisfaction as a result of the friendly and hospitable behavior of caregivers. Whenever I come here, I am treated well. Respectful care is where the caregiver will have patience and spend time on things that matter to the patient.” KB-PC-FG7*

*“Please I have realized that when they finish checking your vitals and blood pressure, they don’t tell you where to sit so sometimes we get confused as where to sit or go next. So for me, I expect them to give further directions after checking our vitals and blood pressure. The processes should be well explained. If not explained well, then I see it as disrespect.” KB-MD-FG7*

*“There are situations where patients are weak and unable to walk. Yet, you may see health workers sitting down and looking at the patient. When you struggle before sitting down, they [health workers] will not ask you about your condition but will rather sit down making calls until they are done with whatever they were doing before attending to you. After asking you, they will even frown and sit there.” SDH-FG4*

*“Respectful care is having interaction with the patient concerning the sickness and giving the patient the needed information and allowing the patient to contribute. It is involving the patient in the care and not ignoring the interest of the patient.” -KB-CH-FG1.*

*“What they did for me is that they explained to me things I do not know and asked me if I have something to say. This shows that they treated me with respect and involved me in their care plan.” -BDH-FG10.*

*“Frankly speaking, most health workers treat patients with disrespect. They don’t know how to talk to patients, be it an elderly or younger patient. So, when health workers are not polite to patients, they are not treating patients with respectful care.”* *BRH-FG6*

*“As my sister said, there shouldn’t be any discrimination. If you go to a church, the first person you meet is the usher. The reception you will receive from the usher will tell you how the church looks like and whether you will stay there or not. The front desk at the clinic serves as the ushering unit of the clinic and their reception matters a lot to us.”* *KB-EY-FG2*

*“In this facility, the first time I came here, I was given a good reception, it was very nice. The nurse directed me nicely and explained to me that they are here because of me, because we are their customers which I think is part of the healing process.” KB-CAD-FG2*

*“Because of the respect I am given at the OPD, it makes me feel that I will get my healing here. The way you talk to us also makes us feel we will be well.”* *KB-CAD-FG5*

*“They take care of us well, right from the OPD to the doctors and nurses, they make us feel fine because of the manner they speak with us. It makes us feel satisfied. In fact, the way the care giver talks and the advice they give to you makes you feel that the person is interested in your recovery.”* *KB-CAD-FG1*

“*…….. Concerning the drug aspect, to me the doctor I saw explained everything to me. He said the medicine is expensive (about GHC1000 and above) and asked if I can afford it. To me I realized he was much concerned about me. He told me what the medicine can do as well.*” *KB-CAD-FG6*

“*I think it is friendly and respectful. My only problem is when it comes to medication, some of the doctors only write it for you without explaining it .” KB-CAD-FG1*

*“In this facility, there is respectful care. I remember when I was asked to go for ECG scan, I didn’t know there even though I come here often but because the facility is big, I didn’t know where to find it. I went to a security man to ask him and he left his post to accompany me to the place, so I see that he has given me respect. The doctor I met was good because of the way she spoke to me and received me. This shows respect is practiced here.”* *KB-AN-FG6*

*“When I came here, I realized that there was respectful care. Doctors patiently explain things to you and give you the help you require. The nurses support a lot in the care of your child. They inform you about what will help your child to receive quick recovery and things that will not help the child. I am happy here.”* *KB-CH-FG1*

*“Long waiting hours. Sometimes you will come early and the doctors will not come on time so you have to wait for long. I am from the Volta region and I have to set off around 1:00 am and arrive early just to be waiting for the doctors. So sometimes it is frustrating.”* *KB-AN-FG7*

*“At the OPD (the records and front desk), they give us numbers, but they hide some of the numbers to be given to some special people of their choice. Three days ago, I saw that those who came late were told that the afternoon shift doctors will take care of them. But all of a sudden, the nurse took a number from the folder and gave it to a certain woman who came in late. So, the patient close to me started complaining then I told the person to exercise patience because it could be that the person’s illness is a very serious one. I went to inform the nurse that next time she should explain certain actions like this to patients so that they don’t get offended. The nurse thanked me for my advice.”* *KB-PC-FG5*

*“They know that there are times that doctors will not be available to attend to you yet they will tell you to come early in the morning and wait for long hours before you are later told to go home because there is no doctor. It hurts so much.”* *KB-MD-FG2*

*“…….. Again, our privacy is not considered because sometimes you can be with a doctor, and while the doctor is attending to you, someone just walks in, which is not the best. They normally do that here, so you need to let them know so that they can put a stop to it.” KB-PC-FG4*

*“Unfortunately, most of the consulting rooms in this facility are open with no consideration to patients’ privacy. All the things and secrets you discuss with the provider may sometimes be heard by other patients. There is no privacy in this facility. I think this is not the best of practice.” KB-EY-FG3*

*“I came here one day and my temperature was measured and it was high. The nurse then spoke to me rudely by yelling at me that my temperature is high so I should go and sit outside. She added that you can’t see the doctor if your temperature is high. I waited outside and even at a point I thought of going home. In fact, I almost wept. At a point, I had the feeling that I should go to surgical to check my temperature. I didn’t know anyone there. I went to meet a nurse there and I asked her to check my temperature and she did and it was down so I returned to the first person. Then she also checked my temperature and saw that it was down so she asked me to go inside. This was during the COVID 19 time.” KB-CAD-FG5*

*“The way they work or how they welcome patients is not usually done right, because of this, there is no respect.” PDH-FG5*

*“Our problem is toilet facilities; we don’t have toilets. We don’t also have bathrooms so we have been doing open defecation. Sometimes you would go and defecate in someone’s farmland and the person would say hey, hey, hey……clear your toilet. At times, you wouldn’t want to clear the toilet with your hands because you have come to the hospital. As for the bathing when you come you have to go to your nearby friend’s house and bath and if your patient’s case is so serious, then she will not bath for several weeks.” BDH-FG5*

*“Please I would like to know if it is compulsory to pay for visiting the urinal outside. Because some patients may not have much money on them. At the hospital where I had my referral, patients are not allowed to pay for using the urinal.” KB-OG-FG2*

*“My problem has to do with where we sit. Sometimes, when you come you see pregnant women standing whilst some husbands and children are rather sitting. When you tell them to excuse you to sit then they will give you cheeky responses. I would be pleased if they are informed about it to allow the patients to sit.”* *KB-OG-FG4*

*“There are times you may forget the clinical processes because you might have visited the hospital for long. So, while finding out about the processes, some of the nurses can be so rude to you.”* *KB-MD-FG3*

***“****There was a time I was asked to do some labs so I asked for direction to the place because I didn’t know the hospital very well. The person who directed me couldn’t direct me very well so I got lost and had to come back to the clinic and when I took a look at the time it was 12:00 pm and was directed to a different place to have the labs. When I returned from the lab to the clinic it was 3:00 pm. In fact, that day I became very tired because of the walking. At the clinic, the person who attended to me also left me and did not inform me that he was done with me. Because what usually happens after you have been attended to is that, you are asked to pay a fee so since that fee was not collected, I assumed that the person has not finished with me. Later, the person came back to apologies for not informing me he was done. Because of the incidence I left here almost at 5:00 pm.” KB-OG-FG2*

*“They should make sure there is medicine in the hospital so that when you bring your patient, they will get medication for the patient. Sometimes, there are some drugs that should not get finished in the hospital but those medicines sometimes get finished in the hospital unless you go and buy them outside the hospital, this is what I also have to say.” PDH-R6*

*“The challenge is that; the hospital lacks medication supply. …… Some of the nurses are selling these medicines so when your patient needs a particular medicine, they will tell you that they have the medicine so you can buy from them.” BDH-R5*

*“Three days ago, after I had been attended to, I wanted to do photocopy and I searched everywhere in Korle Bu but I didn’t get one so I had to go to the road side to do it. This is not fair. It is quite frustrating to me. Korle Bu is a big place so I suggest that at every point we should have a photocopy machine to enable us leave early when we come.” KB-OG-FG6*

*“Respectful care has a positive psychological effect on you the patient. Because it has formed something in your mind in such a way that you feel like this doctor when I see him, the way he will speak with me and comfort me, that alone will heal me. ….., whenever I come and see the doctor the sickness vanishes because of the presence of the doctor.”* *KB-PC-FG4*

*“Respectful care will let you calm down and the pain will reduce. It makes you feel that you are really being treated well and comforted. When you are respected, it means you have been counted as part of human beings.”* *SDH-FG4*

*“With disrespectful care, when you come to the hospital and you are not given the attention you need, you become worried and that alone can make you feel the sickness the more. There was an instance, when I left the OPD to eat because I was hungry. I came back to inquire if my name was mentioned in my absence, and the response I received from the nurse was ‘where were you when your name was mentioned.’ This was said in a rude manner.”* *KB-CAD-FG5*

*“What the health workers are doing definitely creates problems and worries because, it has become the perception of patients that in this hospital whether you come early or not, you will not see your doctor or provider early.” BDH-FG1*

*“When a doctor is treating you with care and respect, it gives you good health and you become satisfied with the service delivery. ……..And if the doctor talks to you and shows you all that you need to do in order to help you, it always makes you feel very happy because the doctor will tell you that if you take the medicine your sickness will go.”* *BDH-FG7*

*“A disrespectful care brings about dissatisfaction so much that at a point I decided not to visit the hospital again and that whatever will happen should happen.” KB-PC-FG7*

*“With respectful care, you always feel like going to the hospital even when you are not well. Whereas when there is no respect or it is a disrespectful care, you feel like not attending the hospital. Because when you feel like when you go, you will be shouted at so I shouldn’t attend.”* *KB-PC-FG1*

*“Respectful care brings cure, comfort and satisfaction with service delivery. You won’t go elsewhere apart from that place you received that respectful care. That’s how come I travel all the way from Kasoa to this facility.” KB-PC-FG6“It affects us negatively and creates much worries because since they don’t treat us well, we will not feel like going to the hospital.”* *BDH-FG5*

**KB-AN-FG1 –** “*My understanding is that, for instance, when you visit a facility and you make inquiries and you are* ***answered not in an annoyance but a polite manner, then it becomes respectful care****.* ***Speaking to us in humility*** *just like what you did when we came in is respectful care. Respectful care is also the situation* ***where you are given a nice reception at a place and you are spoken to very well not shouting at you****”.*

**KB-AN-FG6 –** *“My little understanding is that, when you are not well and you visit the hospital,* ***it is expected that the staff receive you with love, joy, and find out from you what brought you to the hospital.*** *This is a respectful care”.*

**KB-AN-FG2 –** *“Respectful care to my understanding is* ***when you are spoken to well and not in a rude way****”.*

**KB-AN-FG1 –** *“It is just like visiting the hospital to inquire from a staff where to process your card and* ***the staff responded that, ‘’don’t come and disturb me’’, it means the person doesn’t respect you****. I went to a hospital and was given some prescription so I went to give it to an elderly man at the dispensary and he* ***began to shout at me saying ‘’why do you like worrying us too much’’.*** *So, I think such a person* ***does not think about his/her fellow human being****. This to me is disrespect”.*

**KB-AN-FG2 –** *“Disrespectful care is where you are* ***not spoken to properly and the staff does not involve you in any treatment plan or decision****”*.

**KB-AN-FG5 –** *“Sometimes our behaviour change, our body language also speaks a lot. So maybe you wanted to speak to somebody and the way the* ***person behaved begins to put you off****. So, in that case the person has disrespected you and that is my understanding”.*

**KB-AN-FG3 – *“****My understanding is that you have gone to a place you don’t know the terrain and you* ***asked somebody and the person shouts at you.*** *It means the person didn’t show respect to you. That’s my explanation”.*

**KB-AN-FG3 –** *“In my first visit to this facility,* ***I was well received and given the needed directives*** *by the security man at post”.*

**KB-AN-FG5 –** *“I was* ***given the needed attention****. My* ***quarries were resolved and I was happy with that****. Even in my first visit, when I was directed here, I got missing so I asked a security man who* ***left his post and brought me here****. Through that he has been respectful to me and I am glad”*.

**KB-AN-FG6 –** “*In this facility, there is respectful care. I remember when I was asked to go for ECG scan, I didn’t know there even though I come here often but because the facility is big, I didn’t know where to find it.* ***I went to a security man to ask him and he left his post to accompany me to the place, so I see that he has given me respect****. The doctor I met was good because of* ***the way she spoke to me and received me****. This shows respect is practised here*”.

**KB-AN-FG7 –** *“When I came here,* ***everything is in order, there is respect and you are directed well*** *when you want to find out something”*.

KB-AN-FG1 – *“When you visit Korle Bu Teaching Hospital for care, you are made to do so many lab tests. In fact, I initially didn’t know about that. There are certain places in Korle Bu that you wouldn’t know such as this facility. It is quite difficult locating this place. There was a day I was supposed to come here but I didn’t come at the right date. I was supposed to come to anaesthesia clinic before doing the labs. I told the doctor I didn’t know about that so he wrote on my folder that I have been here already but I didn’t know. The clinic had closed but I begged the nurses and I was accepted and assisted me.* ***I became happy because she assisted me.*** *Then I went to the lab technician to take a sample. When I went, she told me she was coming to prick me so I should exercise patient because it will hurt me a bit.* ***I was happy to hear that.***  *There are some places when you go, they won’t even say anything to you before pricking you. They will even shout at you before pricking you****. I become happy for the attention given to me****”*.

KB-AN-FG6 – *“I came here with a different issue. I was booked for surgery before a different issue came up. They told me that the first issue will be done but not now. So, I should do the second one. The doctor wrote a report from surgical and asked me to bring it here. Then the nurse asked that where can she fixed me because they have closed. So, she told me that she can’t allow me to go so she will put me on Monday.* ***Even with what she did it made me satisfied****”*.

KB-AN-FG3 – *“I came on Monday and I was asked by the nurse to rather come on Wednesday. She spoke to me nicely and encouraged me to come early and that I shouldn’t be angry. In fact, the manner in which she spoke to me made me* ***happy because she wasn’t rude to me****”*.

KB-AN-FG6 – *“A nurse who attended to me was very good to me. The nurse assisted and directed me to the place where I can do the Xray and* ***that made me happy***. *When we come and we are* ***harshly handled it triggers our sicknesses and we become worse rather****”*.

**KB-MD-FG4 –** *“When we talk about respectful care it means,* ***where the doctor uses the word ‘please’*** ***whenever he/she wants to speak with you****. You will notice that he/she has showed you respect”.*

**KB-MD-FG2 –** *“It explains* ***how well you are talked to****. For instance, when you approach a nurse to find out something from him/her and* ***he/she shouts at you, it makes you feel sad that you are a sick person****”.*

**KB-MD-FG1 –** *“With respectful care, I see it as* ***trying to have empathy or show concern about someone’s condition****. So that the person can* ***leave the hospital in joy after being attended to****”.*

**KB-MD-FG5 –** *“When we talk about respectful care, it refers to* ***the situation whereby a patient is in need and a doctor/nurse gives the patient the desired attention****.* ***Not that you see the person as a nuisance but rather you show love to the person****”*.

**KB-MD-FG7 –** *“Please I have realized that when they finish checking your vitals and blood pressure,* ***they don’t tell you where to sit so sometimes we get confuse as where to sit or go next****. So, for me,* ***I expect them to give further directives*** *after checking our vitals and blood pressure. The* ***processes should be well explained. If not explained well, then I see it as disrespect****”.*

**KB-MD-FG6 –** *“Disrespectful care can also be in a situation whereby a staff attending to you and checking your vitals ends up* ***pressing his/her phone and also taking “selfie” and also dressing the hair meanwhile he/she is expected to concentrate on the work she is doing****. To me such an action is disrespectful care. Perhaps, the person who has come is a big person from somewhere and* ***your action will put the person off****”*.

**KB-MD-FG2 –** *“At the OPD, our telephone numbers are usually documented. Some of us patients are staying very far, so we expect to be called and informed on time on days a doctor may not be able to see us since an appointment date has been given to the patient.* ***Let’s assume that you are coming all the way from Kumasi, you get here just to be told that the doctor couldn’t come so you cannot be seen****. I think it does not show respectful care”.*

*“When I am coming here, I come very early. There are some people who come to meet you but before you realize they have been seen. There was a man beside me and I asked him why are those people who came to meet us seen earlier? The man replied that here if you want to be seen on a particular day, you need to come a day before to tip those who prepare the cards (records). That was what he told me because he confirmed that he did that. I told him that I can’t pay someone who is being paid.”* *KB-CAD-FG7*

*“I think in my experience,* *our time too is not respected. There was a day I was scheduled to have a surgery so I took my annual leave. I live in the Bono East region of Ghana. I was later called that the surgery has been cancelled and the new date will be communicated but I needed to be reviewed before the surgery will be done. I came for the review and the doctor said, you are going to have the surgery next two days. Meanwhile, I had already cancelled my annual leave and I wasn’t prepared for the surgery at that time. So, I think our time too is not respected.” KB-EY-FG5*

**KB-MD-FG2 –** *“Yes. The doctors I have met here are all correct. The manner they converse with you about the treatment, the advice and directives you will receive will even cause your blood pressure to reduce. So far, the doctors I have met are very good”.*

**KB-MD-FG1 –** *“****I have come to meet four doctors and they all gave me the respect I deserve****. Some of the doctors even* ***gave me that encouragement that this kind of sickness will not kill me****. So, it actually* ***inspired hope in me and I felt at peace****”*.

**KB-MD-FG4 –** *“Honestly speaking about* ***the doctors I have meet here, let’s say three,*** ***they are all good****. The doctors have the* ***passion of attending to patients****. In fact, after you are attended to by them,* ***you feel satisfied. Here they have the patients to take care of patients***”.

KB-MD-FG5- “Some few nurses too are good just as the doctors do for us. They all show respect”

**KB-MD-FG6 –** *“For the doctors I don’t have problem with them but for the nurses,* ***the bad look they will give to you, you wouldn’t like it****. Already you as a patient are frustrated before coming because of your condition.* ***For instance, when you approach them for something the manner in which some of them will talk to you isn’t the best****”.*

**KB-MD-FG5 –** *“As my colleague said* ***as soon as you meet the doctor, you become satisfied****.* ***The last time I came here, when I was leaving to the house, I was very happy because of what the doctor said.*** *Truly, when I got home,* ***I had that encouragement****.* ***I have realised that any time I get to the doctor’s end I am okay but is not so at the nurses’ end****. Our main challenge is from* ***the reception but as soon as you get to the doctor’s end everything is cool****”*.

**KB-MD-FG4 –** *“For me, the nurses who have attended to me are good. You will bear with me that all people cannot be the same, you will surely meet some who will show a negative behaviour”.*

**KB-MD-FG1 –** *“Where I have come to meet disrespectful care is* ***the front desk****. There are instances where after seeing the doctor and the doctor subsequently giving an appointment date for the front desk to do their bookings, you see the front desk personnel rather scolding the patient who is innocent and knows nothing about the booking schedules. They will rather be asking you why you should be given such a date when they know that you are innocent as a patient.* ***The innocent patient is rather blamed so my problem is with the front desk****”*.

**KB-MD-FG3 –** *“There are time you may forget the clinical processes because you might have visited the hospital for long. So, in the course of finding out about the processes, some of the nurse can be so rude to you”.*

**KB-MD-FG5 –** *“As my colleague said, you can’t have all the workers to behave the same, there are some* ***health workers who are good and others are not****. I suggest to you our interviewers that, if you want to fish out the bad ones you can pretend to be sick and come and try them, then you will know the bad ones”.*

**KB-MD-FG6 –** *“What I have also observed is that, when you come as a patient and you don’t know anyone here you are a miserable person. When you come here at dawn, you are likely to go home in the evening. But the way the front desk officers behave you even fear to take their numbers to enable you reach them when you need information whiles you are away. They don’t give you that chance. Please see to our waiting time for us”*.

**KB-MD-FG2 –** *“Today like this I got here at 4:30am, when I checked I was the ninth person but the number I was given was eleventh. Even if they want to favour someone, they should at least* ***consider the people who came early and push those who came late back a bit.*** *Last week, I was here very early in the morning, like somewhere 4:30am and when I was given my card, I was told to go home so it was my daughter (a nurse) who called me to inform me that I am supposed to be attended to since I was here early. Please check that for us”*.

**KB-MD-FG5 –** *“I am from Kasoa and I get here at dawn. They should know that I am here because of a problem not because I have nothing to do. If it hadn’t been because of the problem, I would have been at work.* ***When you are treated that way, you are discouraged and as if you want the person to go home and die****”*.

**KB-SG-FG4 –** *“With a respectful care, it explains* ***how you receive a patient in a nice way and give him/her the necessary care so that the patient, becomes well****. This is a respectful care”.*

**KB-SG-FG2 –** *“I believe that this place is not our home that we just get up and come. We all know that whoever comes here comes because of a problem. I was here last week Tuesday and I was told to come today because Tuesday was not my appointment day. When I came and I asked, I was told not to put my card at the reception when I come because I have paid everything already. Because when I don’t do it that way, I will be asked to pay again so she pinned my card on my folder for me. When they started, I went to show it to the nurse and when I spoke to her, she wasn’t giving me a listening ear at all, she was rude to me. Later, she saw that what she did wasn’t right so she approached me and asked me about my problem. So, I said to myself so this person can talk nicely like this and she had wanted to talk harshly to me?* ***So, I realised that he didn’t show respect to me. It hurts me a little. In fact, what the nurse did initially was a disrespectful care****”.*

**KB-SG-FG 1 –** “*I believe that we all come here because of sickness. There are certain times that the way the person will receive you can even bring down the sickness because of the pampering from the person. What the person will say to you will not make you feel the sickness. They should know that they are here because of us and we are also here because of them. So, if they learn how to communicate to us well it will help us****. Not communicating well to us is disrespectful*”.**

**KB-SG-FG1 –** *“****It is the way the person speaks to you****. The way the person will speak to you will make you feel disturbed before the doctor comes to attend to you. The patient becomes disturbed after being attended to because of the manner he/she was received. Whereas when you are received in a nice way, anytime you are coming to the hospital* ***you are not afraid but when you know you are coming to receive mishandling or yelling to feel inhuman, you will realize that anytime you are coming to the hospital you are already disturbed****”.*

**KB-SG-FGD 6 –** *“Maybe you have come and you have greeted the nurse and didn’t respond and was only doing what he/she is doing without giving you attention. I expect the nurse to attend to you first before continuing with what he/she is doing.* ***Failure to do that implies disrespect****”.*

**KB-SG-FG1 –** *“First of all I thank the management of Korlebu for coming up with this study. I will say that here,* ***majority of the staff talk with humility more than those who do not.*** *It is actually one or two people who do not talk well with patients and I am sure that those people are new in the system so they must be talked to. As my colleague said, when they talk somehow harshly with you and see that they didn’t talk well, they tend to call you back and talk to you well. Here, we thank God that they have respect for the clients”*.

**KB-SG-FG4 –** *“They show respect to us here and they talk well with us”.*

**KB-SG-FG6 –** *“So far everybody gives me respect here. There is respect here.”*

**KB-SG-FG5 –** *“I was administered here for some days after my surgery and during my admission, I was really cared for very well and was very happy. But* ***the only problem I have here is our waiting time which is delayed****”.*

**KB-SG-FG5 –** *“Because of the respect I am given it makes me feel that I will get my healing here. The way you talk to us also makes us feel we will be well”.*

**KB-SG-FG1 –** *“They take care of us well, right from the doctors to all the nurses, they make us feel fine because of the way the person speaks with you. It makes you feel satisfied. In fact, the way the person talks and the advice he/she gives to you makes you feel that the person is interested in your recovery”.*

**KB-SG-FG5 –** *“They do everything well for us. There is no problem. They treat us well”.*

**KB-SG-FG4 –** *“They talk to us well”.*

**KB-SG-FG5 – *“****The time we spend here sometimes is quite worrying. I came here one time and had to sit outside for long and even starting shivering. I have noticed that when we come and we are inside we feel better than the outside. We know that when you are referred to surgical it is the only place that you can receive your care so we also exercise patience so that we can receive our care”.*

**KB-SG-FG6 –** *“For me, I think the doctors are not all that many so that is why we keep long here”.*

**KB-SG-FG4 –** “*I think the doctors should be many so that we can be attend to very fast”*.

**KB-SG-FG1 –** *“From my point of view, when you enter a place like the washroom, it is always neat. With that I will give the hospital 100 percent. They really do well in that area”.*

**KB-SG-FG4 –** “*It is true”*.

**KB-SG-FG6 –** *“There is an improvement because now we don’t sit outside but we are all brought inside so they are doing well”.*

*“Few health workers demonstrate respect to patients. Patients sometimes are afraid to ask for help because some of the caregivers do not talk politely to them. Some delay in attending to the needs of patients. There was a time a patient’s water got finished and a nurse was called to come and attend to her needs. The nurse delayed before attending to the patient. So, where is the respect? If they call you to come and attend to a patient, you have to run and come. That is why I said they don’t show respect.”* *BDH-FG6*

*“Nurses in this hospital don’t show respect because my brother died here and was left there for some time. They were supposed to take him out of that room and report on his death. Instead, they just left him there till evening before the mortuary men came and pick him up. So, I see that nurses in this hospital don’t give respect.”* *BDH-FG1*

*“If a patient is here and no one has time for the person, it is disrespectful care. That is not giving the patient the needed attention.”* *KB-CAD-FG1.*

*“Disrespectful care can also be a situation whereby a staff taking care of you ends up being busy with the phone, taking a ‘selfie’, or dressing their hair. Meanwhile, he/she [health worker] is expected to concentrate on the work.”* *BDH-FG6*

*BDH-R7: The reason why when you go to hospital and they don’t always take care of you properly is when you get to the hospital and you don’t even know where to go for your folder or where to go and take your temperature and where to go for the lab test. The nurses in the hospital are supposed to be showing patients where they would go next when they come to the hospital, but they don’t help the patients to know where to go next when they get the hospital. Some always want to tell you that you don’t know where to go and you are coming here to do what? So, it always makes you a strange person in the hospital, like you are from the south or you are not from this community. They would be talking as if they don’t understand what you are trying to tell them and don’t always listen to you.*

*BDH-R1: What I have to add is that; when you are bringing your patient to the hospital and the patient’s case is so severe and maybe you used a motor king or a Can-do to carry the patient, the way the nurses should have run and come and help you to send your patient inside, they would leave you alone to do what you can do and carry your patient inside for them. So, to me, I am not always happy and I think it is a problem.*

*BDH-R5: To me what I think they are supposed to be doing is when they bring a patient with a motor king or a Can-do, the nurses should see it as their work and always bring their wheel and pick the patient inside. Also, when a patient comes, that patient may be a stranger and would not be able to know where to go for a folder or lab test, temperature, and weight so, the nurse is supposed to show that patient where to go. And they should always take their time and talk to the patient because if you are sick and come, sometimes you would talk to a nurse and the nurse would shout at you and it would cause fear and panic in you. Sometimes you would see some of them as your colleague and want to talk to them but you would be having that kind of fear that they would shout at you and if you don’t talk too, they would quarrel with you that you come and they too are also in their office. So, some of us would come and sit till the evening time and you are not to come and sit till evening time. And the admission side, they can admit a patient and that patient would be crying in pain and the nurses would be sitting. Last night one woman died. This afternoon I bathed and came and a woman was crying in pain for the nurses to just come to check on the woman was a problem. A girl told the nurse to go and check on the woman and the nurse said there is no glass so he cannot risk his life and save the woman so getting to night time the woman became weak and died. So, it is like the nurses don’t take care of us in the way they are supposed to take care of us. And we think it is because of them that we are attending hospital and because of that they also work in the hospital but the way they take care of us we are not happy. So, they don’t take good care of us, they don’t know how to talk to patients. So, I think they should know how to talk to patients this would show that when you receive a visitor in your house you need to know how to talk to that visitor in a good way to show respect to that person so that when that person goes out, she would tell others that oh, this place people have respect and hospitality. So, here our Bongo nurses don’t have respect for us.*

*BDH-R6: What I have is that in this our Bongo hospital if you bring your patients to them, they are always serious with the money part than to check on your patient. Instead of them checking on the patient and after that, they can now tell you your bill to pay, they would rather be asking for money and health insurance. Asking if can you be able to pay the bill. So, if I cannot pay the bill, I should leave my patient to die? What they are doing is not good. Whether the person has money or not just check on the patient and tell the bill and see after that the person will pay or not. You have insurance they would not give you medicine, you don’t have insurance they would not give you medicine and if you go and ask them, they would shout at you where is the medicine! When they prescribed the medicine for you to buy where is it? This is what is happing in Bongo hospital here and it is not good. So, if the nurses want, they should just change because if they are going to continue doing this it would not be fine.*

*BDH-R6: Others don’t show respect only a few of them that show respect. One out of ten is showing respect to patients because they don’t talk politely to people and so when you want to go and ask them something, you would be feeling afraid to ask them. If you tell them that oh this patient’s water has finished, they would delay in attending to you. One girl’s water got finished and they called the nurse to come and check on her the nurse delayed before he came to check on the patient. So where is the respect? If they called you to come and check on a patient, you have to run and come. This is what I said they don’t show respect.*

*BDH-R1: Bongo hospital nurses don’t show respect because my brother who came from Mirigu to this hospital and they transferred him to go to Bolgatanga so he entered where they do heads surgery and died there. And when he died there, they were supposed to take him out of that room before they would go and report that the man is dead, they just left him there till evening time before the mortuary man came and pick him up. So, I see that Bongo nurses don’t give respect.*

*BDH-R4: They don’t give respect because when I arrived, I didn’t know where the scanning room is I entered and asked a nurse and the nurse didn’t even mind me. The nurse was quiet and I waited inside the room for a long time and became tired. So, I went up and asked my mother oh please, I am going to the scanning room but I don’t know where to locate it so she was the one who showed me the scanning room but not the nurses who are working here didn’t show me the scanning room.*

*BDH-R5: What I have to also add is when we are talking about respectful care, this hospital there is no respectful care in it and to talk about the doctors’ side; I remember last year I was sick and came to this hospital and the doctor checked on me and told me I have BP. The doctor asked me; do I have 80 cedis to buy the medicine. And I said I don’t have 80 cedis and the doctor just told me that; you Bongo people it left on to you people, you have a lot of typhoids. you should buy medicine for now if not the cases will too complicated and stop, I don’t have money as Bongo people’s national anthem. So, I was sick and I came to you to treat me and you told me this, I don’t know whether you want me to go and die or what. The doctor might just talk to me this way and my heart will beat and I fall there and die. So, it would mean that it is the doctor who killed me. And because the medicine is for the doctor that is why he sells them. Last year I bought them from the doctor and he gave them to my mother. Last year I brought my mother here and the doctor always asks me to buy the medicine for him to give to my mother. So, the other day he told me that I am suffering from typhoid and he asked me to buy the medicine and I said I don’t have money. It is one monkey that spoils all monkeys but we have other doctors in this hospital who give us much respect. Some of them when you go to them the way they talk to you makes you feel like your sickness has reduced. But some of them their face alone would scare you and give you another sickness and even if he asks you what is wrong with you, you wouldn’t be able to explain yourself well what actually is wrong with you, you would just tell him you have a headache. So, this is why I said they don’t give respectful care but we have some of them that give respect and take good care of patients in this hospital. As for our nurses most of them especially the female nurses don’t just take some of us to be anything. One day a male nurse told a female nurse that the way they are doing one day people will talk about us on radio stations and the female nurse said yes, they should talk she would not risk her life here. So, because they wear uniforms and we don’t wear uniforms that is why they take us to be nothing, they don’t give us respect.*

*BDH-R5: Yes, it is true because the way the doctor would converse with you shows that he loves your life and he wants to help you. When you came, he didn’t talk to you in a way that would let you feel angry. He conversed with you before asking you what is wrong with you and you would also tell him your problem and he gives you medicine to take. I was pregnant and one nurse picked me up to take care of me when I was going to the blood room to take blood, this nurse sent me to the blood room after that, the nurse still used her own motor and sent me to my house. When I gave birth, she couldn’t take me to my house it was my husband that picked me up in the car to the house and when I got to the house, this nurse came and visited me twice. So, this kind of nurse how can you say she doesn’t give respect to people? She gives respect and even does things that you are not expecting she should have done to you. And a nurse who would give you medicine and tells you that be doing this and if God bless you would be okay. This shows that the nurse wants your life. But those who would just tell you to go and buy medicine and would not show you what you would be doing to help you so, you would just be taking the medicine but you won’t know what sickness is in you and if you go back to ask them, they would not take time and talk to you. So that is why I think that the one who would get time for you and talk to you before writing a medicine for you is a person who wants to help you*

*PDH-R5: When someone is ill that person is now like this child and therefore needs to be pampered but when you refuse to pamper that person in order to treat that him or her, you do not have to shout at that person hey, hey, hey if not you cannot treat that person unless you pamper the follow to be able to treat that person so, the respect is for you to pamper that person because that person is in pain and thus not know what he or she does because it is not intentional the whole body is in pain so what do you expect that person to do.*

*R:4 Respect is, maybe I come to the hospital and I do not know where the OPD and lab are located then I ask oh this is where I want to go and as a nurse there you ask me to keep asking people when you are a worker in the hospital, you are expected to help me locate this places rather than telling me to ask people, who will I ask again more than you so you see that if the nurse showed me the places is a sign of respect.*

*PDH-R:6 I see respect and taking care of patients in a way that when you bring your patient the nurses are supposed to rush and meet you to take the person inside and if you want to ask for something they are to take their time and explain things to you for you to be aware of what is going on because are some people who will bring their patient and do not know anything unless they ask to know all these things and when there is the attention it makes the one who brought the sick person to cool down their hearts but when the person comes and ask and no one says anything it means you cannot treat the person.*

*PDH-R:5 Whose who are here are there for the sack of sick people so, when the sick people come, they are to treat them with respect but you can bring someone and everybody is still seated and busy with their phones meanwhile it was due to the work you were able to buy a phone so, they are to respect the sick people because if the sick are not there, there pay will not also be. If you know when you came to work you will not work and every day the mark you but when you are sick no one will take care of you or everyone is busy with the phone, if you were not sick you would have been at home so, when you visit the hospital, l they have to take care of you, respect and welcome you well and treat you so that you can be better.*

*PDH-R:2: You will bring a patient but they do not want to touch that patient meanwhile they also have mothers and fathers at home. The way they should have respected the person so that the one who brought the sick person will cool down the heart knowing the will treat the sick person you brought the are rather looking down upon the person and therefore what they are supposed to do they do not do it but rather sit outside even when you call them they will tell you that they are coming meanwhile they will not come and time will also be running out and if you still try calling them you will end up having a quarrel with them so, this is what most of them do.*

*PDH-R5: Disrespect is when you carry your patient to the hospital, the form of treatment they offer will show that they did not respect you.*

*PDH-R5: The way they will work or how they will welcome the patient is not usually done the right way, because of this there is no respect.*

*PDH-R:3 The phones the press when you bring a sick person, they should know that is a human being you brought and not a goat for you to be more serious on your phone than the sick person, the phone was not the reason why you were here you are to treat sick people when they came and welcome them very well. When the sick person vomits you should know what to do because if it was your father, would you say you will not get close to him just because he vomited you will have to clean him up so when you come and meets someone’s father somewhere and you are responsible to treat him you should know how to handle him to bring out a joyful outcome to you and the one who brought him. this is how I see it.*

*WMH-R1: My child was sick and I brought the child here. One nurse told me to send the child for a lab test and I went to the lab knocked at the door and the nurses refused to come out and perform the test for me. I went there at two o’clock AM and they didn’t come out and one hour time I went back again it was the same story. Five o’clock AM and was still here so, one nurse came and we went there together and knocked at the door but still, they refused to open and perform the test. Around six that we went there again and one male nurse now came out and took the child’s blood for the test. So looking at something like this if not the patient is lucky by now the patient would have died. And something like this if they always respond to the patient earlier, they could detect what is wrong with the patient and save a life. But you wake up at six AM, did you come to work or come to sleep? I know that I can’t work at the hospital that is why I am not here, but you know that you can work at the hospital and you are here and don’t want to do the work again. So, they should know that is because of us that they receive their salaries so they are supposed to have sympathy with us here. Sometimes if you are in pain, you are always frustrated you don’t know where you are. They went for training and they know how to receive and take care of patients so you must be having that kind of skills to be receiving patients and be taking good care of them but they don’t have it even to talk of a little of it in Navrongo hospital. If you want to say something, they will still insult you and add.*

*WMH-R6: The phones, the patient will be in pain but you will see the nurses busy on their phones pressing and if you are talking to them they will not mind you and be pressing their phones. So, it really worrying, they should try and look at it.*

*WMH-R8: I haven’t come to the hospital purposely for my treatment but I have been bringing my children to this hospital and I haven’t experienced respectful care before.*

*WMH-R5: Is only one lady that was here she was respecting patients a lot. When you come and they treat you and discharge you, the woman will come and tell you oh God will help you and your condition become better. But when that woman left this hospital, we haven’t seen such respect given to a patient by a health worker in this hospital before. You are alive or you died they don’t care.*

*WMH-R3: They don’t take care of us as they are supposed to take care of us. That is what I earlier on said a sick person is always frustrated. Like if my child is sick, it is always like myself is sick. Because if you bring your patient to the hospital, the patient will be laying down and doesn’t know where he is and it is always the one who brought the patient to the hospital that normally feels the emotional pains, and in the end, that person will also become confused. And if you go to the nurses to talk to them to come and check on your patient, they talk to you in a way that normally worsens your pains again. So that is why we and the nurses don’t have peace because you are already walking with emotions and the nurses will not also receive your patient with respectful care. If you have a sore and it is not healed and someone hit on it what will happen to you? Blood will definitely come out.*

*WMH-R4: I have never come here to treat myself and the way they received me was not good. I was vomiting and all my body was weak and paining and I was sitting in a cue to see the doctor. When it was up to my turn to enter and see the doctor, I got up and my brother held my hand to support me reached the door when we got to the door to enter, the doctor just shouted at me that they didn’t ask me to come and where I am going, they should send me back so they send me back and I was feeling the pains and the doctor was conversing and laughing with a nurse. I waited for a long and the doctor is calling me so I told my brother to return the folder to the folder room and come and send me to a place where I can get a clinic to manage my condition. So when my brother got up to go then those who are sitting with us were calling him to come back he shouldn’t send the folder. So, when the doctor heard the noise the waiting patients were making, then he now called them to bring me and I said they should leave me to die and if I die, it is not his fault. So they talked to my brother and he sent the folder back and we entered to see the doctor. So, in this hospital, there is someone that will receive you well, and there someone who not receive you well. I don’t know, all of us are human beings but if you meet your colleague is like you have met an animal but not a human being. In truth, we have people that receive patients with respect and we also have someone too if he meets you, doesn’t know that you are a human being and that is what brings the problems.*

*WMH-R4: What the person will do to show that person is giving respectful care is; if he receives the patient and talks to the patient in a respectful manner or words. He can ask you what is wrong with you and you explain to him and he will treat you, give the correct medicine and still wish speedy recovery; he will say oh God will help you and your condition will be treated, and talk to you in a calm manner which will cool your temper and take away fears in you.*

*WMH-R1: What my sister just said is true. When you are sick and someone sympathized with you and talks to you in a calm manner, wishes you a speedy recovery, and says oh God will heal you, this is always medicine for you that can treat your condition. Because I bring my patient to the hospital, and if the nurses tell me oh sister don’t worry just pray to God it will be fine, it will reduce my fear and anger because she has encouraged me so, this alone is even medicine to the patient’s condition.*

*WMH-R2: If you want to tell them about how you are supposed to be treated, it will mean you know the treatment, and if you know the treatment then you should have treated yourself in the house. So, I think you came to someone to treat you so that person knows what he will do to treat you, but if you are going to tell them that they should do here or do this, you will interfere with them and delay their work. They will even tell you that if you know all that then you should treat yourself. So I think when you go to them just describe your condition to them and they themselves know what they will do to treat you.*

*WMH-R4: It is true if you know all that, you wouldn’t have come to the hospital. You should have just treated it yourself. One day I was sick and I went to the hospital in Kumasi the doctor asked me about my problem and I said I have malaria. The doctor said you know that you have malaria and why are coming to the hospital again. So I think the questions that they will be asking just try to answer for them and they themselves will now know what is wrong with you and how they can be able to treat you, you cannot tell them what they should do to treat you.*

*RHB-R7: When they said that this hospital treats patients with respectful care is that they treat patients very well but some of the treatment is not respectful care. We those that are illiterate face a lot of challenges because we don’t know anything, they will just look at you write drug or something for you to go and buy meanwhile you are not supposed to buy and their colleagues will not also look at it and allow you to go and buy. Something small that you are not going to spend much time at the hospital, you will end up spending the whole day there due to illiteracy and no assistance in the hospital.*

*RHB-R3: What they do for us to say that they treat people with respectful care is that when you visit the hospital and a patient cannot walk. The health workers will come and pick up the person take your vitals and take care of the patient very well but some of them don’t treat patients with respectful care.*

*RHB-R6: When they said this health facility treats patients with respectful care, it means they treat patients with respect, care about them, and treat them fine when they visit the facility and when don’t have drugs. The provider will write the drug and inform the client what to do when she/he is able to buy the drug. The hospital will not give patients fake drugs but always make sure that their drugs are very good for treatment.*

*RHB-R1: I will also say that people of old age and pregnant women don’t allow to be joining the queue before seeing the consultant or providers. They normally sit separately so that they will take care of them fast because of their conditions. I also think that is respectful care.*

*R2: When you are sick seriously and visit the health facility. They will run to you and take care of you very well to recover from your sickness.*

*RHB-R: What I will also say to add is that when you come to the health facility the provider or doctor will ask you. What are you suffering from or which part of your body is paining you in a gently or nice way? When you now explain to the provider or doctor, he/she now knows the kind of sickness you are suffering from. The health provider will now write for you to go to the pharmacy for drugs and even if some are not there, she/he will let you go and buy so that the sickness can be treated.*

*RHB-R7: It is really true when you visit a health facility and they treated you with respectful care you normally go to the house with a cheerful face. If you look at respectful care it is good because when you visit the health facility and they treated you like that normally you feel okay before taking the drugs to recover. I am saying this because your child can fall sick and when you visit the health facility, you will see that the health workers are running to welcome you and take your vitals. After that, you will see the provider, and the provider will welcome you well and consult you with respect before giving you the drugs. If all these steps you passed without having any issue with the health providers, then the health facility treats patients with respectful care.*

*RHB-R5: The health workers treated people with respectful care because I fall sick at midnight. When they bring me to the health facility, they woke the health workers on duty up and they came out and treat me very fine when daybreak, I look like nothing happen to me at midnight. The hospital health workers are excellent when it comes to mid-night respectful care treatment.*

*RHB-R6: For, frankly speaking, most health workers treat patients with disrespectful care. They don’t know how to talk to patients especially the old age or younger age. So, when a health worker doesn’t know how to talk to clients cannot also treat patients or people with respectful care too.*

*RHB-R6: I don’t know whether some of them don’t know anything or what because when you come and they ask your name. When you mention it to them, they will ask you to spell the name for them meanwhile it is not all of us that can spell our names, especially the old age. So, if you say that kind of person spells his/her name for you. What can that person do the health workers will become annoyed shouting at the person using words that are not good to use when consulting clients.*

*RHB-R5: What I want to add is that when you come to the hospital and you are suffering instead of them coming and picking you up and communicating well with the patient. They don’t do that and look at the one who brought the patient to the hospital. They will stand there looking for help from the health workers but none of them is willing to welcome them and help. I think that is a problem for us because of the disrespectful care of the health workers.*

*RHB-R4: In the labour ward, I was pregnant and came there to deliver and what happened there, I was saying that if I even give birth at home is better than me coming to the hospital. Instead of them coming and helping me, they were saying that I should come and at that movement, I don’t even know what I am doing and was not even strong to walk again. When I was laying on bed at the hospital, they gave me time for delivery and none of them was willing to check on me again. The child was about to come and a nurse was passing. She was the one who help me to deliver but the ward nurses didn’t check on me. So, looking at that it, they treated me with disrespectful care.*

*RHB-R5: I ever brought my child here in the evening around 8: O’clock PM and no health provider attended to me until 9: o’clock am they said I should go to the laboratory and they will check my child’s blood. I went for them to check and I was there till 12: o’clock pm and no one brought me the results either do they attend to me. My child passed away at the hospital and I back my child home like that and it really pains me a lot.*

*“I think our values are respected anytime we visit this facility but we will not get it done a hundred percent due to some patient’s and health worker’s characters.”* *BDH-FG2*

*“It is not all the time that our choices are respected because sometimes you can tell the providers your choice but they will say that your choice will not work. Sometimes it depends on the kinds of sickness that we are suffering from.”* *SDH-FG7*

*“What they usually do is that anytime I do not understand something pertaining to my treatment they explain things to me and show me what to do for my child. This shows that they treated me with respect and involved me in the care.” BDH-FG10*

*“For today’s treatment the doctor who took care of me, was very good and respectful because he engaged me in communication concerning my health, and life and even extended it to my family members. So, what I will say is that God should richly bless him and his work will improve so that he will continue to treat patients with respectful care and involve them in the treatment plan.”* *SDH-FG5*
